# Whole-drawer imaging for digital management and curation of a large entomological collection

**DOI:** 10.3897/zookeys.209.3169

**Published:** 2012-07-20

**Authors:** Beth Louise Mantle, John La Salle, Nicole Fisher

**Affiliations:** 1 Australian National Insect Collection, CSIRO Ecosystem Sciences, GPO Box 1700, Canberra, ACT, 2601, Australia

**Keywords:** Digitisation, entomology, Whole-drawer, imaging, collections, Satscan

## Abstract

Whole-drawer imaging is shown to be an effective tool for rapid digitisation of large insect collections. On-line, Whole-drawer images facilitate more effective collection management, virtual curation, and public engagement. The Whole-drawer imaging experience at the Australian National Insect Collection is discussed, with an explanation of workflow and examples of benefits.

## Introduction

“Existing taxonomic processes have served us well for centuries but are clearly inadequate for the challenge at hand. The taxonomic community must rally around a common vision……It is time to approach taxonomy as a large scale international science.”

Quentin Wheeler, Peter Raven and Edward O. Wilson

Science, 2004

Libraries of printed material experienced a renaissance in the 1990s when documents were made available in a standardised, portable, digital file format, the PDF. The benefits of producing publications in both physical and digital formats were immediately clear: secure, space-efficient, resource-efficient, economical, accessible, and so on. Arguably, the most important benefit of digitised publications is the ability to search the text within the literature, thus delivering a wealth of previously unknown and/or inaccessible data and information to users.

Natural history collections are libraries of temporal and spatial biodiversity information (Drew 2011). The data in these biological libraries are physically attached to individual specimens and, as a minimum, include information about when and where the specimen was collected, who collected it, and in the case of images what it looks like.

‘Traditional’ digitisation or databasing (i.e. entering label data from, or taking pictures, of individual specimens) of insect collections is inexorably slow, thus large entomology collections must seek alternative, large-scale approaches for improving delivery of biodiversity and taxonomic data to the world (Johnson 2012). Whole-drawer imaging of entomology collections is a digitisation method that is gathering momentum in a number of institutions, including the Australian National Insect Collection (ANIC) (Mantle et al. 2011), the Natural History Museum in London (BMNH) (Blagoderov et al. 2010) and the North Carolina State University (NCSU) Insect Museum (Bertone and Deans 2010). This technique produces high-quality, ultra-high resolution images of whole drawers or trays of insects for online display and extraction of specimen metadata. The resulting images of the specimen (and sometimes associated label) can be viewed, downloaded and annotated, thus providing collections and users with a remote resource for auditing, curating and accessing the collection without physically handling the specimens.

This paper will discuss the whole-drawer imaging project currently underway at the ANIC and provide an assessment against the predicted outcomes for the project. We predict that delivery of high-resolution whole-drawer images will:

1. Promote and encourage remote curation of unsorted specimens;

2. Deliver insect specimen metadata;

3. Assist with loan requests;

4. Provide a method for auditing the collection;

5. Permit morphometric analysis of at least some specimens; and

6. Encourage public engagement with biological collections.

## Materials and Methods

### Equipment

**Figure 1.**
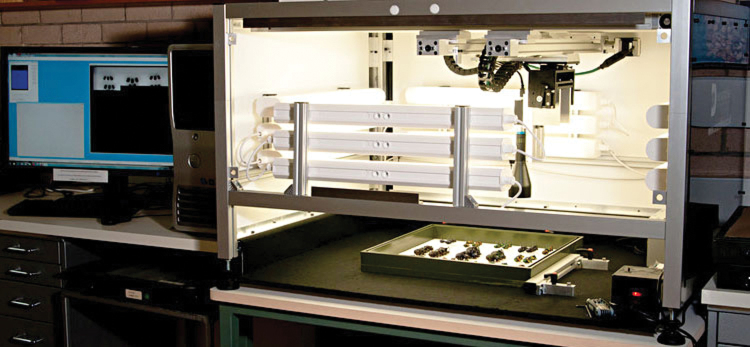
The SatScan imaging system used in ANIC. Shown here with the front cover removed.

Imaging of collection drawers within ANIC takes place by the use of a SatScan™ prototype imaging system (Figure 1), developed by SmartDrive Ltd (http://www.smartdrive.co.uk ). At the time of purchase in 2010 the complete system cost approximately AUD$80–100,000.

The SatScan system uses a combination of hardware and software that automatically captures a series of 200-400 “tile” images at precisely monitored positions. These tile images are then assembled (“stitched”) together to form an extremely high-resolution final image of a drawer of insects.

The ANIC SatScan uses a Basler A631FC 1/2” CCD camera with Edmund Optics 0.16× telecentric lens #NT56-675 that moves in two dimensions along precision rails positioned above the drawer. In this way, the SatScan creates images with minimum distortion, no parallax artefacts and improves the overall coherence of the image. Therefore, all specimens are perfectly imaged with no occlusion from unit tray boxes and with uniform scale so that accurate measurements are valid anywhere throughout the image.

Framework surrounding the camera and lens is clad in a dark plastic material that contains twelve internal fluorescent tube lights for providing adequate light for short exposures (20–40 ms). The framework shields the drawers from surrounding ambient lighting, which could interfere with the controlled illumination inside the SatScan machine. The internal lighting is constant (not flashing) and the system operates quietly so as to not be obtrusive to the working environment.

### Workflow

The SatScan captures sequential “tile” images (200 – 400 per drawer) during working hours, and then automatically “stitches” the tile images overnight to achieve a whole-drawer image. Essentially, the system captures and accurately mosaics together tile images to assemble a single, large image, covering the entire drawer area.

Given an average capture time of 5–7 minutes per drawer, a skilled operator can process up to 60 drawers of specimens each day, and up to 90 final pictures can be stitched in 12 hours (e.g. overnight). These times are typical for a trained operator and bug-free software.

**Figure 2.**
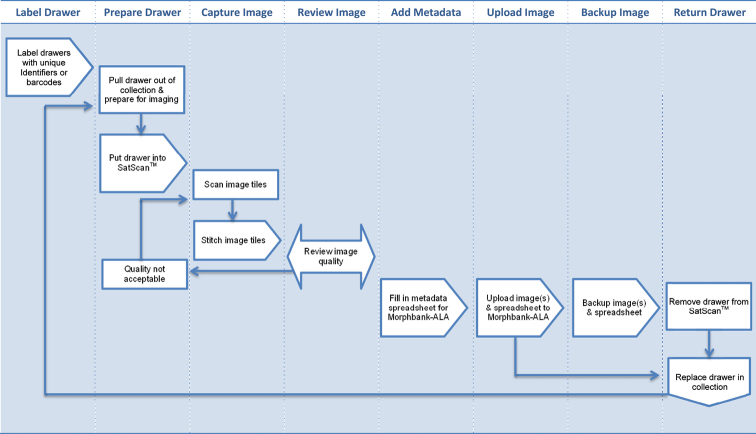
Workflow process in ANIC to Digitise whole drawers of insects and load images into Morphbank-ALA

Each drawer was assigned a unique identifier that also acts as a location code for the drawer within the collection. In addition, the unique identifier is the filename of the image (note – this identifier is not a GUID or LSID and is for internal ANIC use only). Hence, the image file and actual drawer can always be associated together. Figure 2 demonstrates the workflow process for digitisation of whole drawers in ANIC.

Output specifications for imaged ANIC drawers:

• Field of view: 35.5 × 27.5 mm

• Original tile images: 1280 × 960

• Final images: up to 21000 × 21000

• Resolution: ~35 px/mm

• Minimal resolved structures: 0.06–0.1 mm

• Depth of field: 10–80 mm

• File formats: 24bit BMP or LZW-compressed TIFF

• File size (15000 × 14000 px): ~780Mb (BMP), 340Mb (TIFF)

• Exposure: 1–1000 ms

• Capture time of 480 × 500 mm drawers: 5–7 min, depending on exposure

• Stitching time, 200–400 tiles: 5:30–9:30 min

### Image Delivery

Whole-drawer images were uploaded to Morphbank-ALA image repository (http://morphbank.ala.org.au ), where they can be viewed and navigated at a high resolution (i.e. images are zoomable), edited, annotated and shared amongst the collections community, researchers and clients. Morphbank-ALA is a multi-concurrent user, web-based system, supported by all current mainstream browsers. The software is free, open-source and server-based. Images must be imported to make use of the management system, however the next version should enable referencing to externally stored images. Metadata is captured as a DarwinCore record and can be supplemented by additional user defined attributes. The system allocates stable, unique identifiers to images, which can be linked to and referenced in external publications. The system treats images as a representation of a specimen thus the subject in the image is the most important object, not the image itself. Morphbank supports assignment of taxonomic determinations and hierarchy to specimens, it supports groups and role-based security allowing for image collections to be maintained privately, within confined membership groups, and/or published to the public domain.

**Figure 3.**
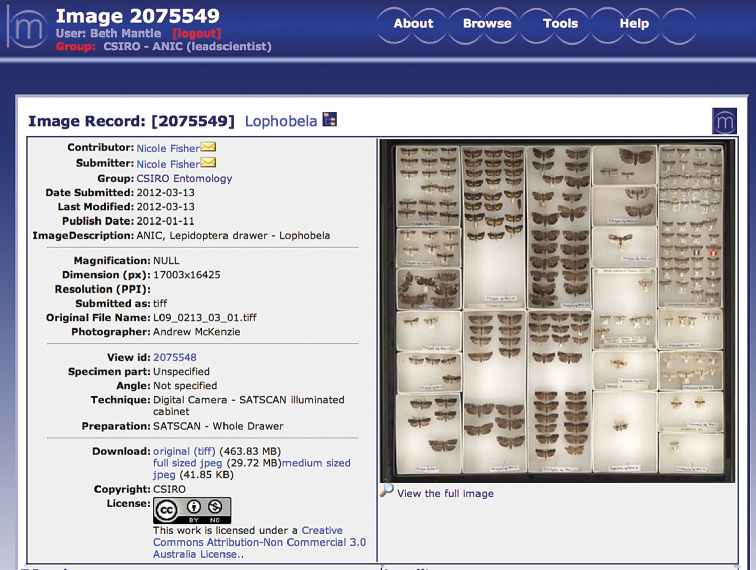
A whole-drawer image displayed in MorphbankALA for online for viewing, editing and download. Image properties: 17,003x16,425 pixels, 30 MB (JPEG), and 464 MB (LZW compressed TIFF).

A typical ANIC entomology drawer measuring 480 × 500 mm produces a final image of 15000 × 14000 pixels, and file size of ~780MB (BMP) or 340MB (TIFF). Figure 3 shows an example of a TIFF drawer image displayed on the Morphbank-ALA website with the persistent URL http://morphbank.ala.org.au/?id=2075549 .

At the time of publication, more than 1,500 whole drawer images were available on Morphbank-ALA. Images can be viewed by browsing the CSIRO-ANIC Group of images.

## Results and discussion

There are many challenges facing collections that plan to digitise specimen data, including: lack of funding support, loss of staff with the expertise required to accurately curate and identify specimens, and difficulty obtaining the appropriate technology and equipment (Vollmar et al. 2010). Some disciplines face greater barriers to digitisation than others. Entomological collections are particularly difficult. Insects are generally mounted on pins with very small labels attached beneath the specimen. To access the data, the specimens must be handled, the label removed from the pin and the associated data decoded and entered into a database. This is equally true for imaging individual specimens. Both forms of digitisation (data-basing, imaging) are time-consuming, and place the specimen at increased risk of damage through handling. Furthermore, entomology collections are large and contain significantly greater numbers of individual specimens than other zoological collections. The Natural History Museum in London (BNHM) boasts 28 million specimens (BMNH website), and the Smithsonian National Museum of Natural History (SNMNH) estimates holdings at more than 35 million specimens (SNMNH website).

The ANIC is the world’s largest collection of Australian invertebrates and is comprised of approximately 12 million pinned, slide-mounted and fluid-preserved specimens. Based on the estimated number of specimens, and the current rate of ‘traditional’ digitisation at the ANIC, it will take a further 250 years to database the entire collection.

Whole-drawer imaging offers a rapid digitisation method that complements traditional databasing and has increased the rate of digitisation at the ANIC. At the time of publication, more than 1,500 collection drawers (from a current total of 22,000 drawers) have been imaged and uploaded to Morphbank-ALA . Although this project is in its early stages, the value of capturing and delivering whole-drawer images online is becoming clear.

### Remote curation of unsorted specimens

Ultra-high resolution images of whole insect drawers provide enough morphological detail to facilitate identification of specimens remotely, which could contribute towards unblocking a significant “bottleneck” in the curation chain (Beaman et al. 2007). The expertise to provide accurate and reliable identifications of particular groups is often unavailable within a collection and therefore specimens cannot be appropriately identified internally. As such, entomology collections rely on visiting researchers to provide identifications and advice regarding reorganization of the collection, in this case by bringing the expertise to the specimens. However, online delivery of whole-drawer images brings the specimens to the expertise, wherever they are located, and increases the opportunity for specimens to reach a useful level of identification.

**Figure 4.**
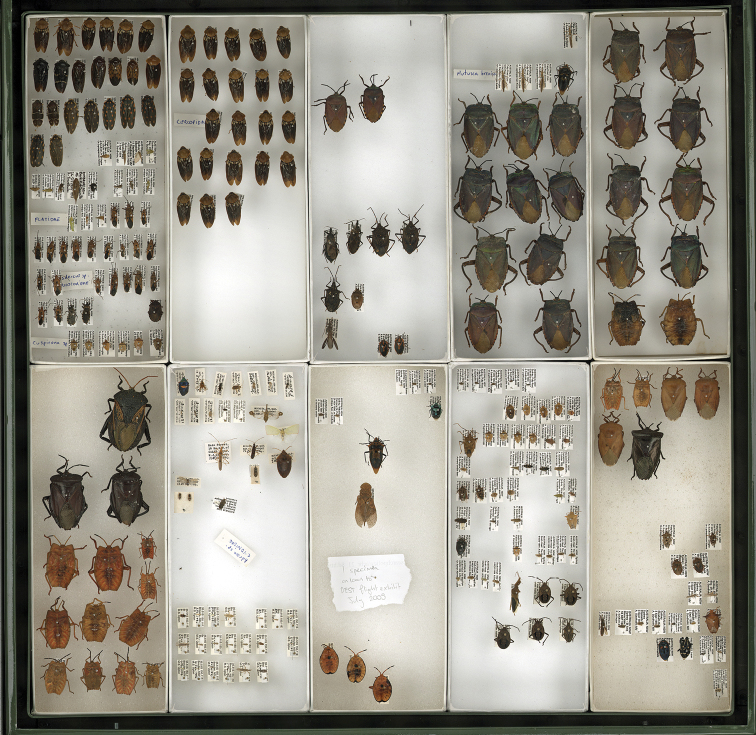
Whole-drawer image of unsorted Hemiptera specimens with identifications provided by a remotely located expert, Dr Murray Fletcher. This drawer was subsequently re-curated according to the identifications, with specimens accessioned into the appropriate locations within the ANIC Hemiptera collection. See Appendix 1 for full list of remote identifications.

For example, an image of an unsorted drawer of Hemiptera specimens (Figure 4) was displayed to illustrate the size and quality of the images produced by the drawer scanner at the annual Australian Entomological Society conference in 2010. Almost immediately, several Hemiptera experts seated in the audience began calling out identifications for the specimens in the image. This exercise demonstrated the potential for remote curation of collections based on identifications of specimens in whole-drawer images.

The level of taxonomic identification using whole-drawer images varies, and is dependent on a number of factors:

1. Size of the specimens. Visual detail of diagnostic characters increases with the size of the specimens being imaged.

2. Complexity of the group. Some specimens will be unidentifiable, regardless of the quality of the image, because the group is geographically, morphologically or behaviourally complex. Non-morphological or non-visual characters, such as internal genitalia, genetics or behaviour, may be required to differentiate many species.

3. Taxonomic understanding of the group. A specimen that belongs to a group that is taxonomically poorly known and/or understood will be difficult to identify to species from an image alone. However, increased levels of curation (e.g. family level to genus level) can be achieved in almost all groups.

Images of drawers from sections of the collection that are being actively curated or revised are at risk of becoming obsolete. The imaging workflow should allow for versioning of images. Furthermore, each drawer is uniquely identified with barcodes so that changes as a result of curation or revision are captured and the drawer is flagged for re-imaging.

### Insect specimen metadata

**Figure 5.**
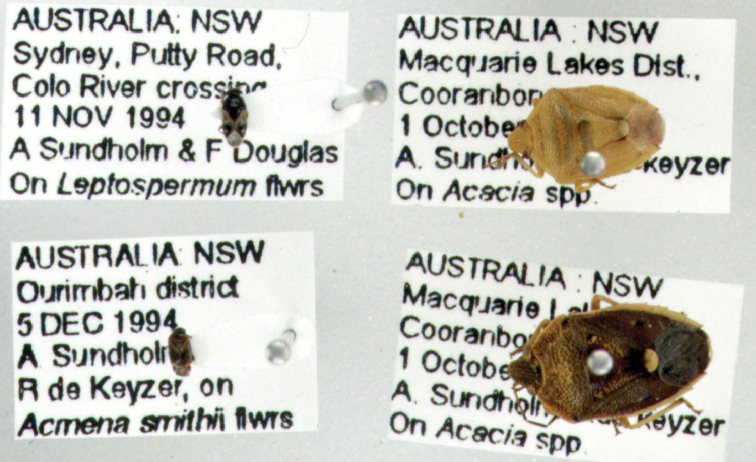
Inset from previous figure (Figure 4). Label data attached to small specimens is often almost completely readable. Therefore, specimen metadata could be extracted and digitised using specialised character recognition software.

Emerging technology that can extract specimen level metadata from images of whole-drawers, specimens and specimen labels will revolutionise digitisation of entomological collections. While whole-drawer images comprised of large specimens may facilitate species identification, images of small specimens have a higher probability of revealing useful and extractable label data. This is illustrated by Figure 4, which shows an unsorted drawer containing both large and small specimens. The small specimens are hard to identify; however, as Figure 5 shows, the labels associated with smaller specimens are almost completely unobstructed from view. It is hoped that, in the future, specialised software will be capable of scanning the image, extracting and recognising the printed text associated with specimens, and automatically creating a searchable database record.

**Figure 6.**
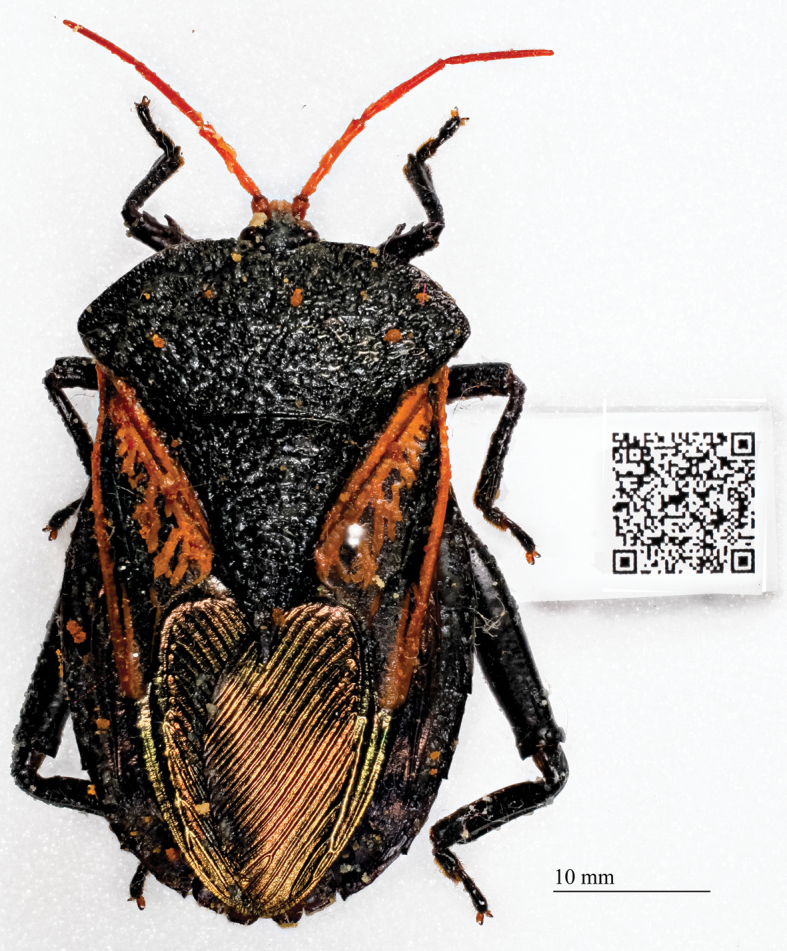
Specimen with QR Code containing label data. A smart phone with the appropriate software can read and access the label data for this specimen from the image.

Specimens for which label data are obscured may benefit from the use of barcodes or QR codes. These codes contain the specimen metadata, are small and thus conserve space in a drawer or unit tray, and can be easily read from the specimen itself, or an image of the specimen, using a smart phone with the appropriate software. Figure 6 provides an example of a QR code attached to a large insect, with label data that can be accessed from an image:

### Loan requests

Requests for loans of material from entomological collections are a resource-intensive process. When a request is received, collections staff assess whether relevant material is available (that is, a significant proportion of the material may be unsorted or unidentified), make value judgements on which material is suitable for loan (for example, damaged specimens would not be acceptable, while type specimens are often excluded from loan requests), complete the appropriate loan and permit paperwork, and securely pack and post the specimens (postage represents a significant expenditure for many large and active collections).

In some cases, the borrowed material does not match the needs of the requestor (for example, the material has been incorrectly identified, or was collected from irrelevant localities). Some loans may consist of up to tens of thousands of individual specimens, requiring days or weeks of preparation.

High-resolution whole-drawer images provide a ‘virtual collection’ for researchers to access and browse for specimens of interest. The images are detailed enough for potential borrowers to judge for themselves if relevant material exists, and whether they wish to request a loan. This delivers a number of savings to the lending institution:

1. Staff are not required to spend time searching the collection for relevant material.

2. If relevant, loanable material is available, the borrower can use a whole-drawer image to indicate precisely which specimens s/he wishes to borrow.

3. Large loans can be accompanied by images of the specimens, negating the need to provide detailed written lists of material on loan forms. This is also useful for tracking overdue loans or partial returns.

**Figure 7.**
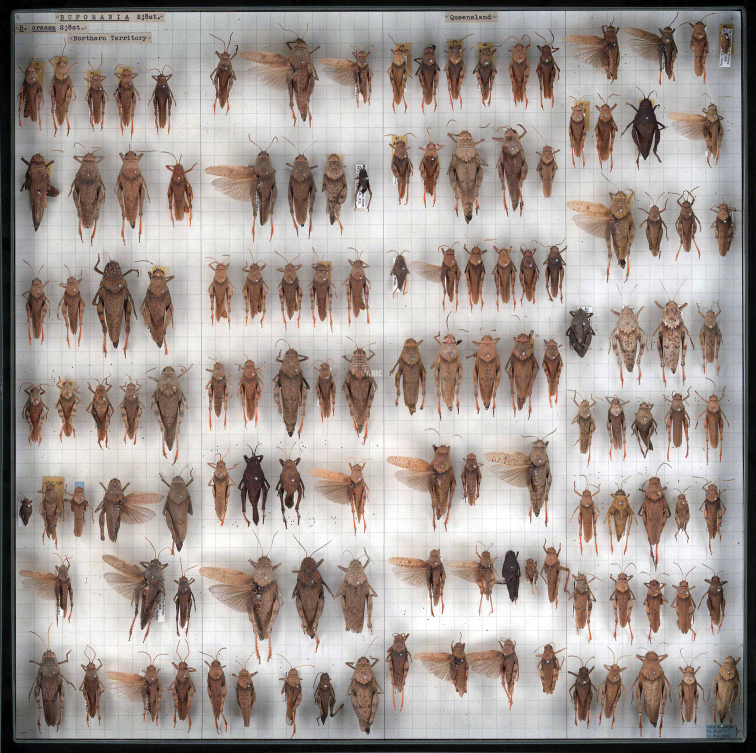
Ultra high-resolution image of Buforaniidae grasshoppers (Orthoptera) from the ANIC. Note that the specimens are arranged by species, and then by the State from which they were collected. In this example, Northern Territory specimens are pinned in the first and second columns, followed by Queensland specimens in columns three and four. The online version of this image is viewable at Morphbank-ALA.

For example, in 2011 the ANIC received an enquiry regarding Buforaniidaegrasshoppers. The ANIC holds 12 drawers of this taxon, which were imaged and provided on-line to the enquirer. Figure 7 shows a curated drawer arranged by species, and then by the State from which the individuals were collected. The enquirer was interested in the geographical distribution of the ANIC specimens; therefore, in this example the whole-drawer images provided all the required information. At this time, no loan was required, no further correspondence was necessary and the whole-drawer images of this group are available online for future enquiries or requests for material.

### Collection auditing

Perhaps unsurprisingly, large entomology collections struggle to develop and implement practical auditing and inventorying procedures. Large numbers of individual specimens (often numbering in the millions) combined with significant gaps in taxonomic knowledge and understanding of invertebrate groups results in a challenging collection management environment. Add to this, continued annual collection growth that may contribute to backlogs of unaccessioned material.

A recent audit of the Australian Museum by the Office of the New South Wales Auditor-General (2010) highlighted three key recommendations: (1) prioritise the collections, (2) tighten inventory control and (3) plan major catch-ups on legacy material. Whole-drawer imaging provides a means for implementing all three of these recommendations.

1. Prioritise the collections.

Resourcing for collection management and development is becoming increasingly limited; therefore, it is critical that the available resources used according to a set of priorities. The Smithsonian Curation Standards and Profiling System (McGinley 1993) assigns a curation standard to individual drawers and is used to calculate a collection health index (CHI). Whole-drawer images provide a means for calculating the CHI and tracking CHI as it changes over time.

2. Tighten inventory control.

Inventory control allows risk assessment in collections. Whole-drawer images can be used to:

• Develop a map of the general locations of specimens in the collection;

• Pin-point specimens that might be considered high-risk (e.g. high monetary value in a commercial market) or high-priority (e.g. holotypes or taxa represented by a single specimen); and

• Create a visual base-line inventory to serve as a basis for future inventory control.

3. Plan major catch-ups on legacy material.

Legacy collection material or backlogs of unaccessioned specimens are at risk from neglect (such as being misplaced or damaged by pests), becoming disassociated from vital collection data (such as field note books), or not being at a curatorial level where they can be made available to experts for revisionary study or further identification. Images of drawers and boxes of legacy material makes specimens “accession-ready” by:

• Improving visibility within the collection, and

• Simplifying the accession process when resources and/or expertise become available.

### Morphometric analysis of specimens.

Measurement of insect morphological characters can be done directly (on a physical specimen using callipers), or indirectly (on an image of a specimen using image analysis software). Direct measurement places specimens at increased risk of damaging through handling and the close proximity of measuring tools. Indirect measurement removes these risks but increases the risk of measurement error due to the positioning of specimens at angles other than perpendicular to the camera lens (projection distortion).

**Figure 8.**
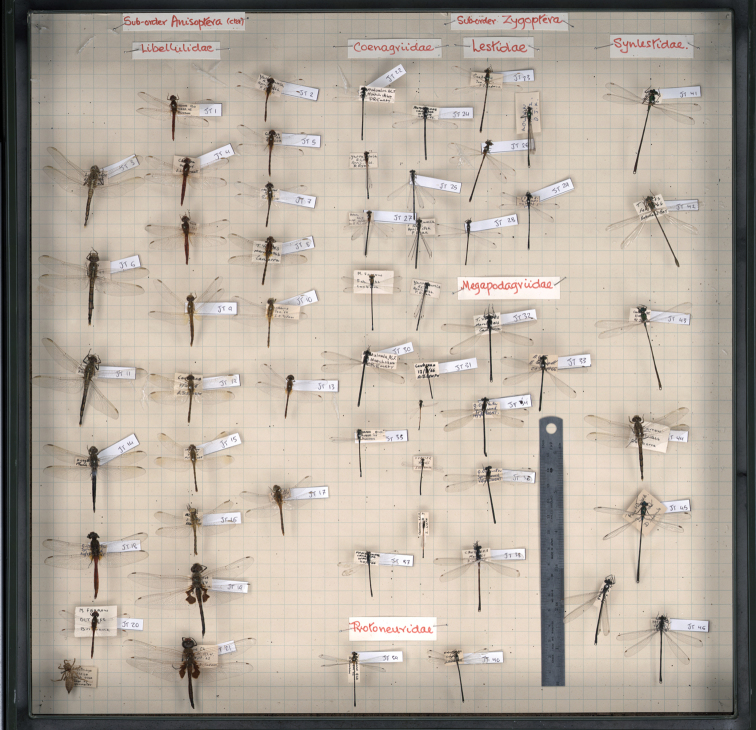
Whole-drawer image of dragonfly specimens used for a pilot study investigating the error associated with direct and indirect measures of morphological characters, such as wing length.

A recent pilot study was conducted in the ANIC to investigate the comparative error rate associated with direct and indirect morphometric analysis of dragonfly wings (Mantle, unpublished data). Wing length of individual dragonflies was measured using three different methods: (1) with callipers on the pinned specimen in the drawer, (2) with callipers on wings that had been dissected from the specimen and slide-mounted, and (3) on a whole-drawer image of the dragonflies (Figure 8).

Preliminary results are encouraging and suggest that, despite variable specimen positioning, there are no significant differences between direct and indirect measures of wing length. In addition, indirect measurement on whole-drawer images was significantly faster (hours rather than days) than measurements taken from individual specimens *in situ*.

### Public engagement with biological collections.

Drawers of curated insect specimens elicit wonder and delight from members of the community. Some institutions can capitalise on the community’s fascination with insects through public exhibitions and educational programs. The ANIC, however, is a research-only facility that does not have front-of-house, or public displays. Delivering high-resolution whole-drawer images of some of the most visually attractive specimens from the collection may:

• Improve public engagement with the research activities of the collection;

• Increase the collection’s profile within the broader community; and

• Provide a platform for delivery of virtual education and outreach services.

Furthermore, opportunities exist to collaborate with, and add value to, existing online public resources. For example, whole-drawer images illustrating various insect families could be linked to the “What Bug Is That?” interactive key (http://anic.ento.csiro.au/insectfamilies/ ) and to galleries of insect taxa in the Atlas of Living Australia (www.ala.org.au ). Crowd-sourcing is another initiative used to actively engage the community in natural history collections by facilitating the digitisation of insect collections through online “volunteer portals” (see http://volunteer.ala.org.au/ ).

## Conclusions

High-resolution whole-drawer imaging of the ANIC specimens has been beneficial to both the collection and its users. The project is improving curation and auditing processes by providing a mechanism for tracking specimens through space and time. Engagement with researchers has improved because the metadata available from whole-drawer images adds value to correspondence about specimens. Consequently, the imaging project will continue and it is estimated that every drawer will be available for viewing online by 2015.

## Acknowledgements

The authors would like to thank the ANIC imaging volunteers: Andrew McKenzie, Emily O’Connor, Fiorella Esquivel and Frederick Michna for their assiduous efforts in preparing and imaging the insect collection drawers; Laura Johnson for the dragonfly images and measurements; Peter Brenton for assistance with delivering the images and associated metadata through Morphbank-ALA; and Murray Fletcher for identifications of Hemiptera. We are grateful to the Atlas of Living Australia for providing funding to support the purchase of equipment for this project. We also thank Matt Bertone, Stefan Schmidt and Vladimir Blagoderov for their critical reading and valuable comments in improving the contents of the paper.

## Appendix 1

List of identifications provided by Dr Murray Fletcher based on high-resolution Figure 4. Identifications presented in the following order:

Unit trays 1–5 in the upper row, left to right;

Unit trays 6–10 in the lower row, left to right;

Rows 1–n from top to bottom in each unit tray, left to right along each row; and

Individual specimens separated by a comma.

**Table:**

**Box 1**	
Rows 1–3	Large Cercopidae from Malaysia
Row 4.	2 as above, ?, possibly Neuroptera, poss. Neuroptera, ?
Row 5.	?, ?, ?, *Amarusa australis* (Jacobi) (Cercopidae: Aphrophorinae), ?, ?, ?, ?
Row 6.	Flatidae, ?, Membracidae, Heteroptera, Heteroptera, Heteroptera
Row 7–10.	all Heteroptera
**Box 2**	
	All large Malaysian Tessaratomidae
**Box 3**	
Row 1.	2 × Tessaratomidae
Row 2.	Reduviidae, Reduviidae, Pentatomidae, Pentatomidae
Row 3.	Pentatomidae, Pentatomidae
Row 4.	Alydidae, 2 × *Agonoscelis rutila* (Pentatomidae)
**Box 4**	
Row 1.	*Mutusca brevicornis* (Alydidae) according to the label
Row 2.	3 *Mutusca brevicornis*, + 1 scutellerid
Row 3–6.	all large Tessaratomidae
**Box 5**	
	All large exotic Tessaratomidae
**Box 6**	
	All large exotic Tessaratomidae, lower ones are nymphs
**Box 7**	
Row 1.	Scutelleridae, Heteroptera, Heteroptera, Heteroptera, ?, ?, ?
Row 2.	?, ?, ?, ?, ?, *Thanatodictya* sp (Dictyopharidae)
Row 3.	Flatidae (possibly *Colgar* sp.)
Row 4.	Alydidae, Heteroptera, Scutelleridae
Row 5.	Achilidae: Plectoderini, ?
Row 6.	?, ?, ?
Row 7–9	lots of little things. Last one in Row 9 might be *Dascalina* or *Massila* (Flatidae)
**Box 8**	
Row 1.	?, ?, ?
Row 2.	?, ?, Heteroptera
Row 3.	Scutelleridae
Row 4.	Ledrini (not Australian)
Row 5.	3 × Tessaratomidae nymphs
**Box 9**	
Row 1.	?, ?, ?, ?, ?
Row 2.	4 × Pentatomidae, ?
Row 3.	5 × Pentatomidae, ?, ?
Row 4.	?, Membracidae, Heteroptera, Heteroptera, Auchenorrhyncha
Row 5.	4 × Pentatomidae, Iassini, ?
Row 6.	?, ?, ?, Pentatomidae
Row 7.	Pentatomidae, Pentatomidae, ?, ?, ?, ?
Row 8.	?, ?, ?, ?, Heteroptera, Pentatomidae, Pentatomidae
Row 9.	3 × Pentatomidae
**Box 10**	
Rows 1–2.	Tessaratomidae
Row 3.	2 × Pentatomidae
Row 4.	2 × Pentatomidae
Row 5.	2 × Heteroptera
Row 6.	Heteroptera, ?, ?, ?, ?, ?, Pentatomidae
Row 7.	Scutelleridae, Pentatomidae, ?, ?
Row 8.	?

## References

[B1] Polaszek A Alonso-Zarazaga M Bouchet P Brothers DJ Evenhuis NL Krell FT Lyal CHC Minelli A Pyle RL Robinson N Thompson FC van Tol J (2005) ZooBank: the open-access register for zoological taxonomy: Technical Discussion Paper. Bulletin of Zoological Nomenclature 62: 210-220

[B2] Beaman R Macklin JA Donohue MJ Hanken J (2007) Overcoming the digitization bottleneck in Natural History Collections: A summary report on a workshop held 7-9 September 2006 at Harvard University. http://www.etaxonomy.org/wiki/images/b/b3/Harvard_data_capture_wkshp_rpt_2006.pdf [Accessed 29 February 2012]

[B3] Bertone MA Deans AD (2010) Remote curation and outreach: examples from the NCSU Insect Museum GigaPan Project. In: Proceedings of the First International Conference on Gigapixel Imaging for Science, November 11–13 2010.

[B4] Blagoderov V Kitching I Simonsen T Smith S (2010) Report on trial of SatScan tray scanner system by SmartDrive Ltd. Available from Nature Precedings http://hdl.handle.net/10101/npre.2010.4486.1 [Accessed 25 February 2012]

[B5] Drew J (2011) The role of natural history institutions and bioinformatics in conservation biology. Conservation Biology 25 (6): 1250-1252 doi: 10.1111/j.1523-1739.2011.01725.x 22070276 10.1111/j.1523-1739.2011.01725.x

[B6] Johnson N (2012) A collaborative, integrated and electronic future for taxonomy. Invertebrate Systematics 25: 471-475 doi: 10.1071/IS11052

[B7] Mantle BL Fisher N La Salle R J (2011) Whole drawer imaging for curation and management of the ANIC. TDWG 2011 Conference, New Orleans, Louisiana, USA. http://www.tdwg.org/fileadmin/2011conference/slides/Mantle_SatScanANIC.pdf [Accessed 15 March 2012]

[B9] McGinley RJ (1993) Where’s the management in collections management? Planning for improved care, greater use, and growth of collections. International Symposium and First World Congress on the Preservation and Conservation of Natural History Collections Vol. 3: 309-338

[B10] New South Wales Auditor-General’s Report (2010) Knowing the Collections: Australian Museum Performance Audit. http://australianmuseum.net.au/document/Knowing-the-Collections-audit-report [Accessed 15 March 2012]

[B11] Vollmar A Macklin JA Ford L (2010) Natural history specimen digitization: challenges and concerns. Biodiversity Informatics 7: 93-110

[B12] The British Natural History Museum Entomology Department webpage: http://www.nhm.ac.uk/research-curation/departments/entomology/ [Accessed 2 March 2012]

[B13] The Smithsonian National Museum of Natural History Department of Entomology wepage: http://entomology.si.edu/ [Accessed 2 March 2012]

[B14] The Australian National Insect Collection webpage: http://www.csiro.au/en/Organisation-Structure/National-Facilities/Australian-National-Insect-Collection/ANIC-Profile.aspx [Accessed 2 March 2012]

